# Use of Ketotifen Fumarate-Eluting Daily Disposable Soft Contact Lens in Management of Ocular Allergy: Literature Review and Report of Two Cases

**DOI:** 10.7759/cureus.27093

**Published:** 2022-07-21

**Authors:** Junji Ono, Hiroshi Toshida

**Affiliations:** 1 Ophthalmology, Ono Eye Clinic, Shizuoka, JPN; 2 Ophthalmology, Juntendo University Shizuoka Hospital, Shizuoka, JPN

**Keywords:** pollinosis, allergic conjunctivitis, ketotifen, anti-allergic drug, contact lens

## Abstract

We present two cases in which the drug-eluting daily disposable soft contact lens (DDSCL) Acuvue® Theravision^TM^ with Ketotifen (Johnson & Johnson Vision Care, Inc., Jacksonville, Florida, USA) (ATK), which contains the anti-allergic drug ketotifen fumarate, alleviated ocular allergic symptoms. Case 1 was a 57-year-old woman with a history of allergic conjunctivitis in the spring and fall. In the summer and winter, the patient used frequent replacement soft contact lenses, but in the allergy seasons, she used DDSCLs in combination with anti-allergic eye drops. She agreed to start wearing ATK lenses from before the next fall season. The lenses suppressed her allergic signs and symptoms, and she felt comfortable wearing them and continued their use throughout the season. Her symptoms were suppressed, providing her comforts that enabled uses of the CLs for some time. She successfully used the lenses again from the beginning of the next spring season. Case 2 was a 31-year-old woman with a history of cedar pollinosis. The patient previously used DDSCLs and was prescribed ATK lenses at her regular clinic. About one month later, she was referred to our department. With ATK lenses, she did not experience any subjective symptoms during the pollinosis season. At a subsequent visit, hyperemia had decreased compared with the initial visit. In these two cases, the use of ATK lenses from before until the end of both the spring and fall allergy seasons suppressed the symptoms of allergic conjunctivitis, allowing the patients to continue to wear contact lenses.

## Introduction

Acuvue® Theravision^TM^ with Ketotifen (Johnson & Johnson Vision Care, Inc., Jacksonville, Florida, USA) (ATK) is the first drug-eluting contact lens (CL) containing an anti-allergic drug. It was first launched in Canada in 2021 [1] and was approved by the Pharmaceutical and Medical Devices Agency (PMDA) in Japan in March 2021. The product was then launched at select eye clinics in Japan in fall 2021. ATK was approved in Japan not as a drug, but as a drug-eluting daily disposable soft contact lens (DDSCL) for the correction of visual acuity. The lens material is etafilcon A, which is a copolymer of 2-hydroxyethyl methacrylate and methacrylic acid cross-linked with 1, 1, 1-trimethylolpropane trimethacrylate and ethylene glycol dimethacrylate. Water content is 59% and oxygen permeability is 21.4 x 10^-11^ (cm^2^/sec) (mL O_2_/mL x mmHg) at 35°C. Diameter is 14.2 mm and the base curve is 8.5 mm and 9.0 mm [2]. These data are similar to one-day Acuvue®. Each lens contains 0.019 mg of ketotifen fumarate. When the lens is applied to the eye, the drug is released and enters the lacrimal fluid [1]. According to the United States Food and Drug Administration report, the lenses are indicated for the prevention of ocular itch due to allergic conjunctivitis and correction of refractive ametropia in patients who do not have red eye(s) [2].

About half of people in Japan have allergic conjunctivitis, including allergic conjunctivitis without papillary proliferation and seasonal allergic conjunctivitis (SAC) [3,4]. Every early spring, there is a marked increase in the number of patients with allergic conjunctivitis due to pollen from cedar or cypress trees [5,6]. Patients have itchy eyes and hyperemia and symptoms associated with allergic rhinitis. In the fall, many patients show similar symptoms due to ragweed and wormwood pollen [7].

Here, we describe the successful use of ATK in patients with spring cedar pollinosis and fall ragweed pollinosis.

## Case presentation

Both patients had a history of allergic conjunctivitis without any non-ocular symptoms or systemic complications. All procedures performed in studies involving human participants were in accordance with the ethical standards of the institutional committee and with the 2018 version of the Declaration of Helsinki [8] and its later amendments or comparable ethical standards. This case report was approved by the review board of Shizuoka Hospital, Juntendo University (No. 862).

Case 1

**Figure 1:**
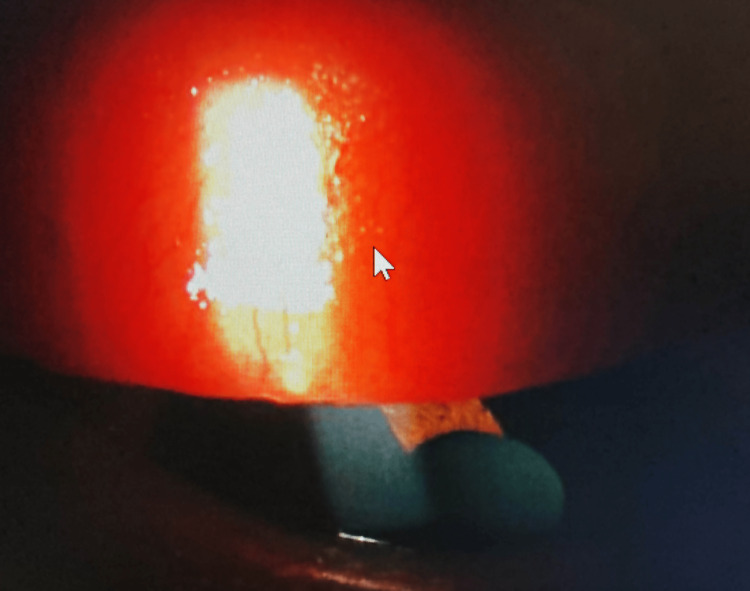
Palpebral conjunctival findings of the upper eyelid at the initial visit. Hyperemia and papillary proliferation (arrow) were seen in the left eye before prescribing ATK.

Case 1 was a 57-year-old woman who experienced ocular allergic symptoms every spring and fall. Subjective symptoms included itchy eyes and eye discharge. In addition, frequent replacement soft CLs (FRSCLs) tended to move upwards because of papillary proliferation of the palpebral conjunctiva. The prominent findings were hyperemia of the upper eyelids and papillary proliferation on both sides at the initial visit to our hospital (Figure 1).

The patient used FRSCLs but was switched to DDSCL before every allergy season from 2004. The CL-corrected visual acuity was 0.9 in both eyes. Before the fall allergy season in 2021, the patient visited us and asked to be switched to DDSCL before her symptoms reoccurred. We explained the characteristics and potential risks of ATK to the patient, who consented to treatment and started to use the lenses.

**Figure 2:**
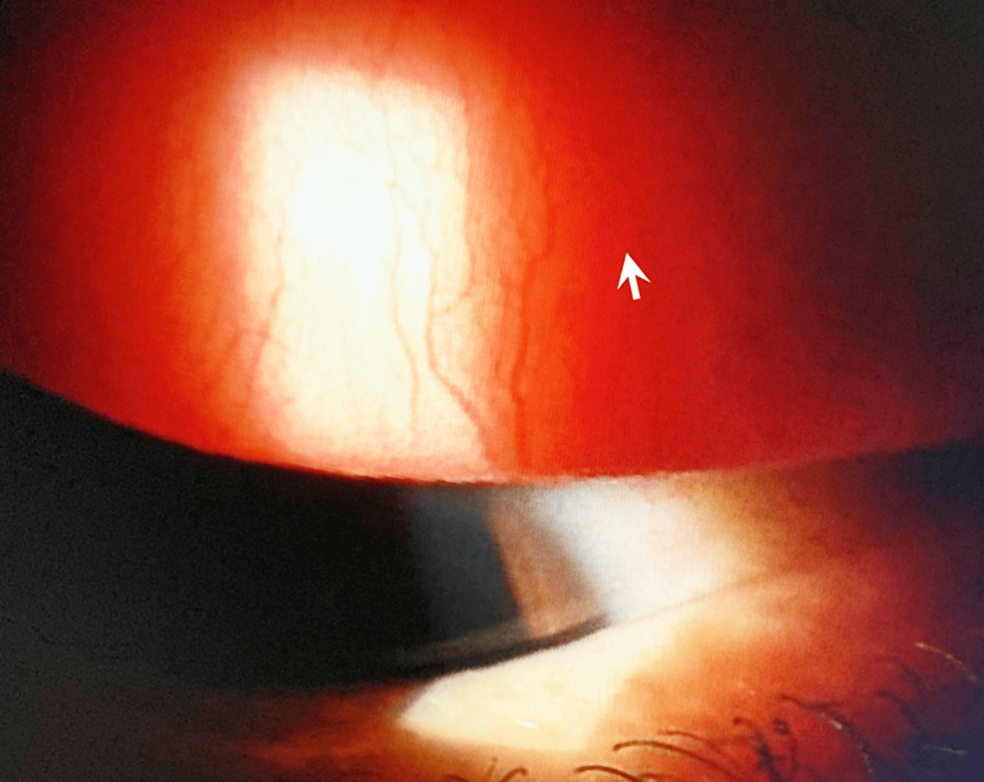
Palpebral conjunctival findings of the same eye as Figure 1 after using ATK. Hyperemia and papillary proliferation (arrow) were decreased comparing to Figure 1.

First, the patient wore the ATK lenses during visits to the hospital on three trial days. Her visual acuity was 0.9. Four days after the final trial day, the patient visited us again. Because ATK had favorable clinical effects, it was prescribed for one month. The patient visited our facility 1.5 months later, which was 15 days later than the originally scheduled visit. She stated that she had had no symptoms while wearing ATK. Once she had used up her supply of ATK after a month, she started to use FRSCLs, which resulted in the onset of itching. She also mentioned that she had used anti-allergic eye drops. She reported no subjective symptoms, presumably because of the eye drops. The patient was prescribed ATK again. Figure 2 shows findings of the palpebral conjunctiva of the same eye as Figure 1. Few hyperemia or papillary proliferation was seen. Because both the subjective symptoms and the wearing conditions were favorable, the patient continued to use ATK until the end of the fall allergy season, when she switched back to an FRSCL. Half a year later, the patient used ATK during the spring pollinosis season and did not experience any subjective symptoms or ocular complications. The patient continues to use ATK.

Case 2

**Figure 3:**
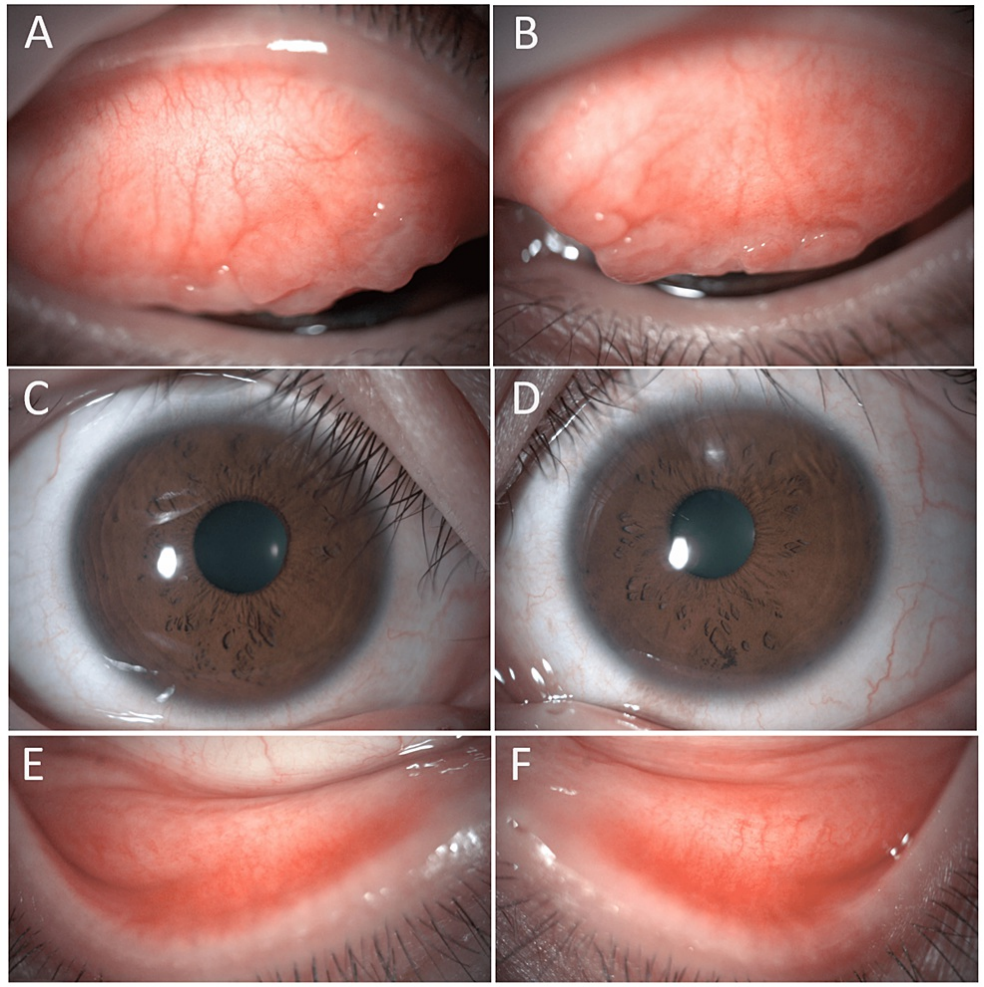
The ocular findings before the patient started wearing ATK. Conjunctival papillary proliferation of the upper eyelids (A: right eye, B: left eye), bulbar conjunctiva (C: right eye, D: left eye) and mild hyperemia of both the upper and lower eyelids (E: right eye, F: left eye) were shown in both eyes.

**Figure 4:**
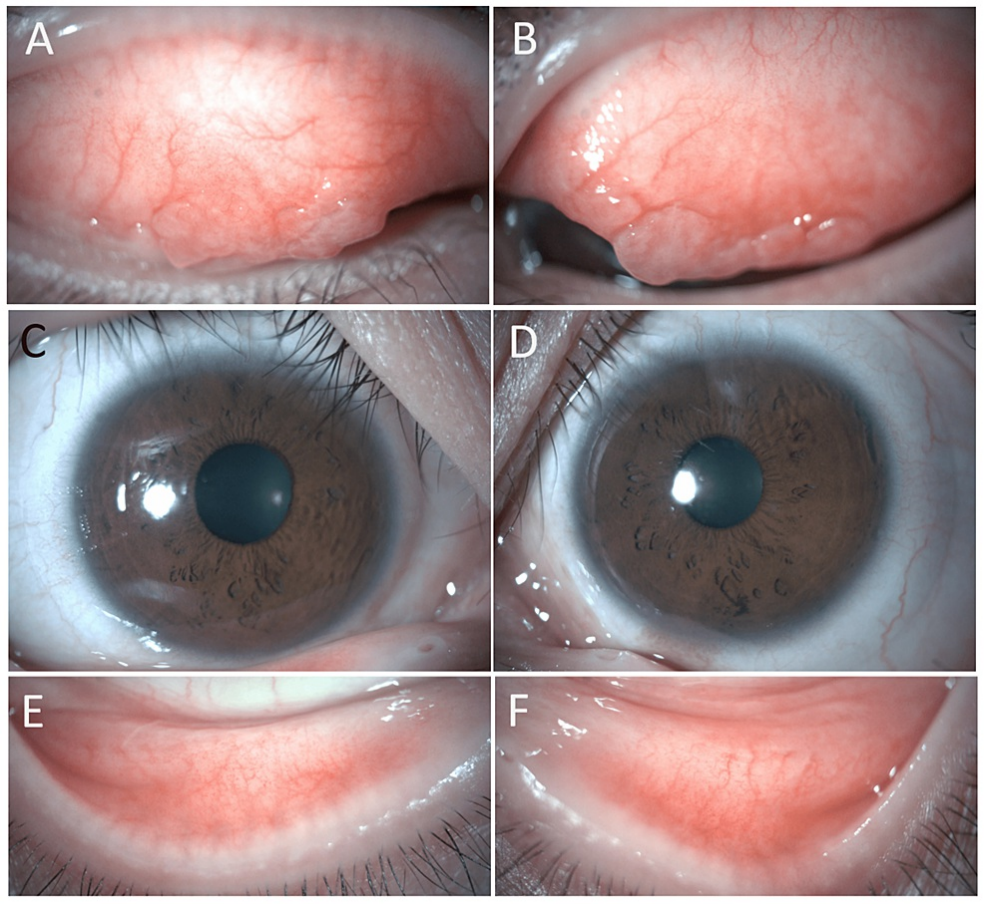
The ocular findings after using ATK for one month on the spring pollinosis season. Hyperemia of the upper eyelids (A: right eye, B: left eye), bulbar conjunctiva (C: right eye, D: left eye) and the lower eyelids (E: right eye, F: left eye) had decreased. There was no worsening of conjunctival papillary proliferation of the upper eyelids in both eyes (A: right eye, B: left eye).

Case 2 was a 31-year-old woman who experienced itchy eyes and eye discharge due to cedar pollinosis in the spring. The patient previously used DDSCLs and was prescribed ATK lenses at her regular clinic. About one month later, she was referred to our department. With ATK lenses, she did not experience any subjective symptoms during the pollinosis season. At a subsequent visit, hyperemia had decreased compared with the initial visit. Figure 3 shows a photograph of the anterior eye before the patient started wearing ATK. At that time, she did not have subjective symptoms. However, an examination showed conjunctival papillary proliferation of the upper eyelids and mild hyperemia of the lower eyelids of both eyes. Figure 4 shows a photograph of the anterior eye after the patient had worn ATK for one month and spring pollinosis season had started. There was no worsening of the conjunctival papillary proliferation of the upper eyelids, and hyperemia of the lower eyelids had decreased. The patient did not report any subjective symptoms such as itching or discharge. Two months after wearing ATK, the pollinosis season ended, and the patient temporarily discontinued the use of ATK. No ocular complications have occurred since then.

## Discussion

Many CL users with allergic conjunctivitis experience ocular allergic symptoms such as itching, hyperemia, and eye discharge while wearing CLs [9,10]. However, lens manufacturers do not recommend using eye drops while wearing CLs [11,12]. Pharmaceutical companies do not recommend eye drops containing benzalkonium hydrochloride (BAK) on top of CLs except on Hard CLs. Therefore, ATK was developed to correct visual acuity and alleviate allergic symptoms in patients with allergic conjunctivitis who wear CLs [1,13,14]. Because ATK is a CL, it must be prescribed by an ophthalmologist. A single ATK lens contains only 0.019 mg of ketotifen fumarate, which is less than or comparable to the amount in over-the-counter eye drops. Accordingly, there was some concern that ATK may have insufficient anti-allergic effects. However, the two cases presented here demonstrate that ATK suppresses allergic symptoms and alleviates hyperemia. We hypothesize that ATK is effective because it releases the ketotifen fumarate gradually and because the drug accumulates in the lacrimal fluid below the CL, so the duration of action is longer than after administration by instilling eye drops.

The ATK lens is negatively charged and takes up the positively charged ketotifen fumarate. After the ATK lens is placed on the eye, ketotifen fumarate is released into the lacrimal fluid. Moreover, because the pH of ketotifen fumarate is similar to that of lacrimal fluid, the ATK lens does not cause any discomfort. In addition, ATK is free from BAK. The two patients reported here did not report feeling a burning sensation when wearing ATK, but the author did for the first few seconds; however, the feeling disappeared quickly. Some people feel a burning sensation after instilling over-the-counter eye drops with ketotifen, so the burning sensation immediately after inserting ATK may be due to the ketotifen.

The ATK lens manufacturer recommends that patients with allergic conjunctivitis start to use the lens before the onset of ocular allergic symptoms. They also recommend that if symptoms appear, use of the lens should be discontinued, and treatment of allergic conjunctivitis should be prioritized. The lens is contraindicated in people with allergic symptoms for whom the lens is not suitable and those with hypersensitivity to ketotifen. While wearing this lens, instillation of ketotifen-containing eye drops is not recommended. Moreover, one lens is used for each eye per day. To date, no adverse effects of ATK have been reported. Thus, although there are several precautions for the use of ATK, this lens has the following benefits for people who need correction of visual acuity by CL and alleviation of allergic symptoms: 1) alleviation of allergic symptoms if worn from before the start of the allergy season; 2) no risk of failure to instill eye drops; 3) avoidance of affecting the lens by instilled eye drops; 4) no risk of preservative containing in the eye drops; and 5) prevention to damage to both their ocular surface and their lenses caused by eye-rubbing [15]. In the two cases presented here, symptoms of allergic conjunctivitis were suppressed in both the spring and fall by using this CL before the start of each allergy season, and both patients could continue to wear the CL.

According to the clinical guideline issued by the Japanese Society of Ocular Allergology, the first-line therapy for allergic conjunctivitis in Japan is the instillation of anti-allergic eye drops [3]. The drops should be started two weeks before the expected start of pollen shedding or once symptoms occur. This approach is expected to reduce symptoms during peak pollen shedding. On the other hand, CL users treat pollinosis by using daily replacement SCL if the itching goes away and, if compatible with the CL, instilling BAK-free anti-allergic eye drops before and after use of the CL. ATK is now available as an alternative treatment approach. If patients with ocular allergic symptoms start to wear the ATK lenses before the allergy season, they can continue to wear them throughout the season and do not have to wear glasses instead of CLs [11,14]. In the two cases presented here, the ATK lens suppressed the onset of allergic symptoms during the spring and fall pollinosis seasons in Japan, and the patients could continue to wear the lens. Moreover, no ocular complications were observed. In case 2, conjunctival hyperemia was reduced. Because of the limited number of patients, further follow-up is warranted on adverse effects and ocular complications. However, the use of this contact lens is expected to benefit many patients with allergic conjunctivitis.

## Conclusions

The use of ATK which is a ketotifen fumarate-eluting DDSCL before the start of allergic season alleviated ocular allergic symptoms in patients with seasonal allergic conjunctivitis. It seems that this lens has the advantages to reduce risks concerned with the instillation of eye drops including preservatives and prevent damages to both their ocular surface and their CLs caused by eye-rubbing. The appearance of ATK may make it possible to continue wearing CLs during the allergic season in patients with seasonal allergic conjunctivitis.
